# Characterization of the complete mitochondrial genome of *Lysmata vittata* (Decapoda: Hippolytidae)

**DOI:** 10.1080/23802359.2021.1930215

**Published:** 2021-06-02

**Authors:** Jinhui Chen, Changhua Xian, Yuehong Luo, Manfen Lin

**Affiliations:** Food and Drug College, Qingyuan Polytechnic, Qingyuan, China

**Keywords:** Mitochondrial genome, phylogeny, Hippolytidae

## Abstract

*Lysmata vittata* is widely distributed in the Indian and Pacific oceans. In this study, we sequenced the mitochondrial genome of *L. vittata* using Illumina HiSeq. The complete mitochondrial genome of *L. vittata* was 20,837 bp in length, including 13 protein-coding genes, 22 transfer RNA genes, and two ribosomal RNA genes. The contents of the four bases in the mitochondrial DNA were A (31.68%), T (32.36%), C (21.65%), and G (14.31%). Phylogenetic analysis of 41 shrimp showed that *L. vittata* was clustered with other Hippolytidae shrimp.

The red-striped shrimp *Lysmata vittata* (Decapoda: Hippolytidae) is a popular shrimp in the marine aquarium trade (Marin et al. 2012). It is widely distributed in the Indian and Pacific oceans, including along the coasts of Japan, China, Indonesia, Australia, and eastern Africa (Wang and Sha 2018; Alves et al. 2019). The species is commonly found at depths of 2 to 50 m and lives in large groups among rocks, algae, and sponges (Alves et al. 2019). Research on this species remains limited, although several studies have investigated its mating, larval development, gonadal development, and diet (Yang and Kim 2010; Chen et al. 2019). The complete mitochondrial genome (mitogenome) of *L. vittata* is an important resource for evolutionary research. In this study, we report on the complete mitogenome of *L. vittata* and analyze its phylogenetic relationships within Hippolytidae.

Specimens (voucher no. QP20200526-1) were collected from the South China Sea (22°36′ N, 114°32′ E), Shenzhen, Guangdong Province, China, and were stored in the herbarium of Qingyuan Polytechnic (Guangdong, China). Muscle samples of *L. vittata* were dissected and preserved at −80 °C until use. The muscle tissue was used for mitochondrial DNA (mtDNA) extraction with a TIANamp Marine Animals DNA Kit (Tiangen, Beijing, China) according to the manufacturer’s specifications. The mtDNA was sequenced using Illumina HiSeq (Illumina Inc., San Diego, CA, USA). Clean data were acquired and assembled using SPAdes v3.15.2 (Bankevich et al. 2012). MITO (http://mitos.bioinf.uni-leipzig.de/index.py) (Bernt et al. 2013) and ORF finder (https://www.ncbi.nlm.nih.gov/orffinder/) were used to identify and annotate protein-coding, transfer RNA (tRNA), and ribosomal RNA (rRNA) genes. Phylogenetic analysis was conducted using maximum-likelihood (ML) in MEGA X (Kumar et al. 2018).

The mitogenome of *L. vittata* was 20,837 bp in length (GenBank accession number: MW285083.1), which is larger than that of other species belonging to the *Lysmata* genus. This may be due to the 4-kb noncoding sequence between cox1 and cox2 in the *L. vittata* mitogenome, which is much longer than that of other species of *Lysmata*. The tRNA-Leu divided this noncoding sequence into two sequences.One was 1712 bp length with A + T content of 71.67%, and the other was 2270 bp length with A + T content of 72.82%. The *L. vittata* mitogenome contained 13 protein-coding, 22 tRNA, and two rRNA genes. Of the 37 genes, 22 were encoded by the heavy strand and 15 genes, including four protein-coding (*ND1*, *ND4*, *ND4L*, and *ND5*), two rRNA, and nine tRNA genes, were encoded by the light strand. The A, G, C, and T contents of the heavy strand were 34.36%, 11.74%, 17.03%, and 36.87%, respectively, with a high A + T content of 71.24%. All protein-coding genes had ATN as the start codon. Ten protein-coding genes (*ND1*, *ND2*, *ND3*, *ND4L*, *ND6*, *Cox2*, *Cox3*, *ATP8*, *ATP6*, and *Cytb*) contained a TAA stop codon, one protein-coding gene (*Cox1*) contained a TAG stop codon, and two protein-coding genes (*ND4* and *ND5*) contained an incomplete T– stop codon. 16S rRNA and 12S rRNA were 1 467 bp (72.26% AT content) and 821 bp (67.07% AT content) in length, respectively. All tRNA genes ranged from 51 to 77 bp in size.

Based on the 13 complete concatenated protein-coding genes of 41 shrimp from the GenBank database, a phylogenetic tree was constructed using the maximum-likelihood (ML) method and Jones-Taylor-Thornton (JTT) matrix-based model, with a bootstrap of 1 000 replicates (Figure 1). The phylogenetic tree showed that *L. vittata* was clustered with *L. amboinensis*. However, *Saron marmoratus* and *Rhynchocinetes durbanensis* were clustered together. This result was similar to the results of previous research (Terossi et al. 2017; Wang et al. 2021), which all proved that Hippolytidae can be considered as a polyphyletic taxon. In conclusion, our study described the complete mitogenome of *L. vittata* and analyzed its phylogenetic position within Hippolytidae. This research should contribute to further investigations on the molecular evolution and conservation of this species.

**Figure 1.**
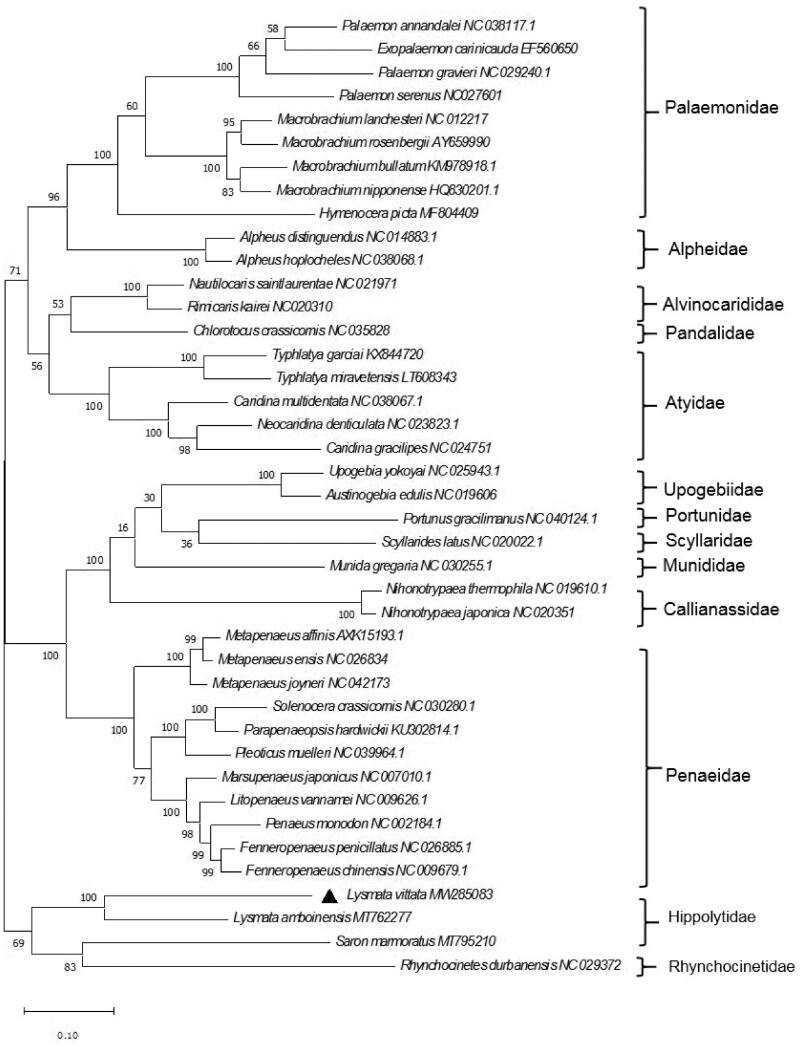
Phylogenetic tree of *L. vittata* and related species based on maximum-likelihood (ML) method.

## Data Availability

The data that support the findings of this study are openly available in NCBI at https://www.ncbi.nlm.nih.gov/, reference number MW285083.

## References

[CIT0001] Alves DFR, López GL, Barros-Alves SP, Hirose GL. 2019. Sexual system, reproductive cycle and embryonic development of the red-striped shrimp *Lysmata vittata*, an invader in the western Atlantic Ocean. PLoS One. 14(1):e0210723. 30645636 10.1371/journal.pone.0210723 PMC6333369

[CIT0002] Bankevich A, Nurk S, Antipov D, Gurevich AA, Dvorkin M, Kulikov AS, Lesin VM, Nikolenko SI, Pham S, Prjibelski AD, et al. 2012. SPAdes: a new genome assembly algorithm and its applications to single-cell sequencing. J Comput Biol. 19(5):455–477. 22506599 10.1089/cmb.2012.0021 PMC3342519

[CIT0003] Bernt M, Donath A, Jühling F, Externbrink F, Florentz C, Fritzsch G, Pütz J, Middendorf M, Stadler PF. 2013. MITOS: improved *de novo* metazoan mitochondrial genome annotation. Mol Phylogenet Evol. 69(2):313–319. 22982435 10.1016/j.ympev.2012.08.023

[CIT0004] Chen D, Liu F, Zhu Z, Lin Q, Zeng C, Ye H. 2019. Ontogenetic development of gonads and external sexual characters of the protandric simultaneous hermaphrodite peppermint shrimp, *Lysmata vittata* (Caridea: Hippolytidae). PLoS One. 14(4):e0215406. 31002693 10.1371/journal.pone.0215406 PMC6474713

[CIT0005] Kumar S, Stecher G, Li M, Knyaz C, Tamura K. 2018. MEGA X: Molecular Evolutionary Genetics Analysis across computing platforms. Mol Biol Evol. 35(6):1547–1549. 29722887 10.1093/molbev/msy096 PMC5967553

[CIT0006] Marin IN, Korn OM, Kornienko ES. 2012. The caridean shrimp *Lysmata vittata* (Stimpson, 1860) (Decapoda: Hippolytidae): a new species for the fauna of Russia. Russ J Mar Biol. 38(4):359–363.

[CIT0007] Terossi M, De Grave S, Mantelatto FL. 2017. Global biogeography, cryptic species and systematic issues in the shrimp genus Hippolyte Leach, 1814 (Decapoda: Caridea: Hippolytidae) by multimarker analyses. Sci Rep. 7(1):6697. 28751634 10.1038/s41598-017-06756-1 PMC5532279

[CIT0008] Wang Y, Zeng L, Wen J, Li X, Huang Y, Sun Y, Zhao J. 2021. Characterization of the complete mitochondrial genome of *Saron marmoratus* (Hippolytidae, Decapoda) and its phylogenetic analysis. Mitochondrial DNA B Resour. 6(1):124–126. 33490599 10.1080/23802359.2020.1848474 PMC7808751

[CIT0009] Wang YR, Sha ZL. 2018. Description of two new species of *Lysmata* Risso, 1816 (Decapoda, Lysmatidae) from the China seas, with remarks on *Lysmata vittata* (Stimpson 1860). Zootaxa. 4392(1):28–40. 29690415 10.11646/zootaxa.4392.1.2

[CIT0010] Yang HJ, Kim CH. 2010. Zoeal stages of *Lysmata vittata* (Decapoda: Caridea: Hippolytidae) reared in the laboratory. Korean J Syst Zool. 26(3):261–278.

